# Depth Estimation from Light Field Geometry Using Convolutional Neural Networks †

**DOI:** 10.3390/s21186061

**Published:** 2021-09-10

**Authors:** Lei Han, Xiaohua Huang, Zhan Shi, Shengnan Zheng

**Affiliations:** School of Computer Engineering, Nanjing Institute of Technology, Nanjing 211167, China; xiaohuahwang@gmail.com (X.H.); shiz@njit.edu.cn (Z.S.); zhengsn@njit.edu.cn (S.Z.)

**Keywords:** depth estimation, deep learning, light field, EPI, convolutional neural network, textural image

## Abstract

Depth estimation based on light field imaging is a new methodology that has succeeded the traditional binocular stereo matching and depth from monocular images. Significant progress has been made in light-field depth estimation. Nevertheless, the balance between computational time and the accuracy of depth estimation is still worth exploring. The geometry in light field imaging is the basis of depth estimation, and the abundant light-field data provides convenience for applying deep learning algorithms. The Epipolar Plane Image (EPI) generated from the light-field data has a line texture containing geometric information. The slope of the line is proportional to the depth of the corresponding object. Considering the light field depth estimation as a spatial density prediction task, we design a convolutional neural network (ESTNet) to estimate the accurate depth quickly. Inspired by the strong image feature extraction ability of convolutional neural networks, especially for texture images, we propose to generate EPI synthetic images from light field data as the input of ESTNet to improve the effect of feature extraction and depth estimation. The architecture of ESTNet is characterized by three input streams, encoding-decoding structure, and skipconnections. The three input streams receive horizontal EPI synthetic image (EPIh), vertical EPI synthetic image (EPIv), and central view image (CV), respectively. EPIh and EPIv contain rich texture and depth cues, while CV provides pixel position association information. ESTNet consists of two stages: encoding and decoding. The encoding stage includes several convolution modules, and correspondingly, the decoding stage embodies some transposed convolution modules. In addition to the forward propagation of the network ESTNet, some skip-connections are added between the convolution module and the corresponding transposed convolution module to fuse the shallow local and deep semantic features. ESTNet is trained on one part of a synthetic light-field dataset and then tested on another part of the synthetic light-field dataset and real light-field dataset. Ablation experiments show that our ESTNet structure is reasonable. Experiments on the synthetic light-field dataset and real light-field dataset show that our ESTNet can balance the accuracy of depth estimation and computational time.

## 1. Introduction

Estimating depth information is a crucial task in computer vision [1]. Many challenging computer vision problems have proven to benefit from incorporating depth information, including 3D reconstruction, semantic segmentation, scene understanding, and object detection [2]. Recently, depth from the light field has become one of the new hotspots, as light-field imaging captures much more information on the angular direction of light rays compared to monocular or binocular imaging [1]. The plenoptic cameras such as Lytro and Raytrix facilitate the data acquirement of a light field. Refocusing images, sub–aperture images, and epipolar plane images (EPIs) can be generated from the light field data. Many new methods of depth estimation have emerged based on these derived images. Especially, EPI-based depth estimation is more popular.

EPIs exhibit a particular internal structure: every captured scene point corresponds to a linear trace in an EPI, where the slope of the trace reflects the scene point’s distance to the camera [3]. Some methods have obtained depth maps by optimizing the slope metric of straight lines in EPIs, and standard feature metrics include color variance, 4D gradient, structure tensor, etc. It is challenging to model the occlusion, noise, and homogeneous region using feature metrics, so the accuracy of these methods is limited. Furthermore, the global optimization process is always computationally expensive, which hampers its practical usage.

With the rise of deep learning, some efforts have integrated feature extraction and optimization into a unified framework of convolutional neural networks, achieving good results. These advances are due to the feature extraction capability of deep neural networks. The research shows that convolutional neural networks are very good at feature extraction of texture images [4]. However, the current depth estimation methods based on deep learning seldom directly use rich texture features in EPIs. Moreover, some methods use complex network structures with many parameters and less consideration of the computational cost. Taking EPINet [5] as an example, it shows good performance against the HCI (Heidelberg Collaboratory for Image Processing) benchmark. However, the depth map resolution obtained by EPINet is lower than that of the central view image. It is not wholly pixel-wise prediction or lightweight.

In this paper, we focus on designing a novel neural network that directly utilizes textural features of EPIs based on epipolar geometry and balances depth estimation accuracy and computational time. Our main contribution is twofold:

- EPI synthetic images: We stitch EPIs row by row or column by column to generate horizontal or vertical EPI synthetic images with more obvious texture. The two EPI synthetic images, as well as the central view image, are used as the multi-stream inputs of our network. In this way, a convolutional neural network (CNN) can play an essential role in texture feature extraction and depth-estimation accuracy. As far as we know, our work is the first to use EPI synthetic image as the input of a depth estimation network. In terms of multi-stream inputs, our network is significantly different from EPINet [5], which takes the sub-aperture image stack as the input of each stream, whereas we use EPI synthetic images.
- New CNN architecture for end-to-end lightweight computing: We employ skip-connections to fuse structural information in shallow layers and semantic information in deep layers to reduce our network parameters and computational time. Furthermore, transposed convolution modules are used to improve the resolution of the output disparity map in order to be consistent with the central view image, thus forming an end-to-end training model and cutting down training complexity.

As an extended version of our conference paper [6], this paper enriches the principle description and experimental verification. The remainder of the paper is organized as follows. Section 2 reports related studies on depth estimation using EPIs from the light field. Section 3 describes the geometric principle and synthetic texture used in our method. Section 4 details our network architecture, including the general framework, multi-stream inputs, skip-connections, and the loss function used in training. Section 5 presents the experiments performed and discusses the results. Finally, Section 6 concludes this paper.

## 2. Related Work

In the following, we briefly introduce existing approaches, focusing our description on the light-field depth estimation methods using EPIs. According to different technical principles, EPI-based depth estimation methods can be divided into two types: EPI analysis and deep learning.

EPI analysis-based methods extract depth information from the light field by evaluating the directions of the lines in EPIs. The idea is to try out all the different directions: the one with the least color variance along the line is most likely to give the correct depth value. Based on this point, several methods use different ways to measure color variance. Kim et al. employed a modified Parzen window estimation with an Epanechenikov kernel [3]. Tao et al. [7] used the standard deviation to measure correspondence cues, then combined this with the defocus cue to calculate depth. Since all EPI data has a similar gradient pattern, it is unnecessary to try out all hypothetical depth values to find the optimal. Accordingly, Mun et al. [8] efficiently reduced the number of angular candidates for cost computation.

Similarly, Han et al. [9] select only eight sub-aperture images with different directions to compute stereo disparity and fuse stereo disparity and defocus response, based on guided filtering, to produce high-quality depth maps. Other EPI-analysis-based methods employ gradient or a structural tensor. For example, Wanner and Goldluecke [10] applied the 2D structure tensor to measure the direction of each position in the EPIs. Li et al. [11] used the depth estimation from the structure tensor as a starting point, followed by a refinement step based on examining the color correspondence along the detected line from the structure tensor. To reduce the computational complexity associated with match cost functions, Neri et al. [12] make a local estimation based on the maximization of the total loglikelihood spatial density aggregated along the epipolar lines. Using epipolar geometry, Lourenco et al. [13] first detect enlarged silhouettes, then devise a structural inpainting method to reconstruct the disparity map. Li and Jin [14] propose a novel tensor, Kullback-Leibler Divergence (KLD), to analyze the histogram distributions of the EPI’s window. Then, depths calculated from vertical and horizontal EPIs’ tensors are fused according to the tensors’ variation scale for a high-quality depth map. Through EPI analysis, Schilling et al. [15] integrate occlusion processing into a depth estimation model to maximize the use of the available data and obtain general accuracy and quality of object borders. Jean et al. [16] and Lin et al. [17] use frequency domain information and focus stacks on estimating depth, respectively. Some studies extend gradient and tensor analysis to 4D space. Berent et al. [18] apply a segmentation technique to identify the 4D plenoptic structures and consequently the depths. Lüke et al. [19] encoded depth information in the “slopes” of the planes in 4D ray space that correspond to a point in the 3D world, so an eigenvalue analysis on the 4D local structure tensor is performed to distinguish types of structure.

Recently, deep learning-based methods continue to emerge. Heber et al. [20] explored a convolutional neural network to predict the 2D hyperplane orientation in the light-field domain, corresponding to the depth of the 3D scene point. Heber also formulated a convex optimization problem with high-order regularization. From this point of view, Heber’s CNN is not an end-to-end network for depth estimation. Guo et al. [21] also disentangled a complex task into multiple simple sub-tasks, and a tailored subnetwork realized each subtask. Finally, an occlusion-aware network was proposed for predicting occlusion regions accurately. In 2017, Herber et al. [22] presented a U-shaped regression network involving two symmetric parts, an encoding and a decoding part. This network unifies ideas from 2D EPI analysis with spatial matching-based approaches by learning 3D filters for disparity estimation based on EPI volumes. To enhance the reliability of depth predictions, Shin et al. [5] design a multi-steam network that encodes each epipolar plane image separately. Since each epipolar plane image has its unique geometric characteristics, the multi-stream network fed with different images can take advantage of these characteristics. However, the output resolution of this network is smaller than that of sub-aperture images, which inconveniences subsequent applications such as 3D reconstruction. Liang [23] proposed EPI-refocus-net, a convolutional neural network that combines EPI cue and refocusing cue for depth estimation. Zhou et al. [24] introduced a hybrid learning architecture to combine multimodal cues from multiple light-field representations. Ma et al. [25] proposed a novel end-to-end network (VommaNet) to retrieve multi-scale features from reflective and texture-less regions for accurate disparity estimation

## 3. Geometric Principle and Texture Synthesis

Different from the traditional camera, the light-field camera adds a microlens array (MLA) between the sensor and the main lens. Through the main lens and MLA, the ray recorded by the light-field camera includes not only the position but the direction. Light-field imaging geometry and data lay the foundation for light-field depth estimation.

**Figure 1:**
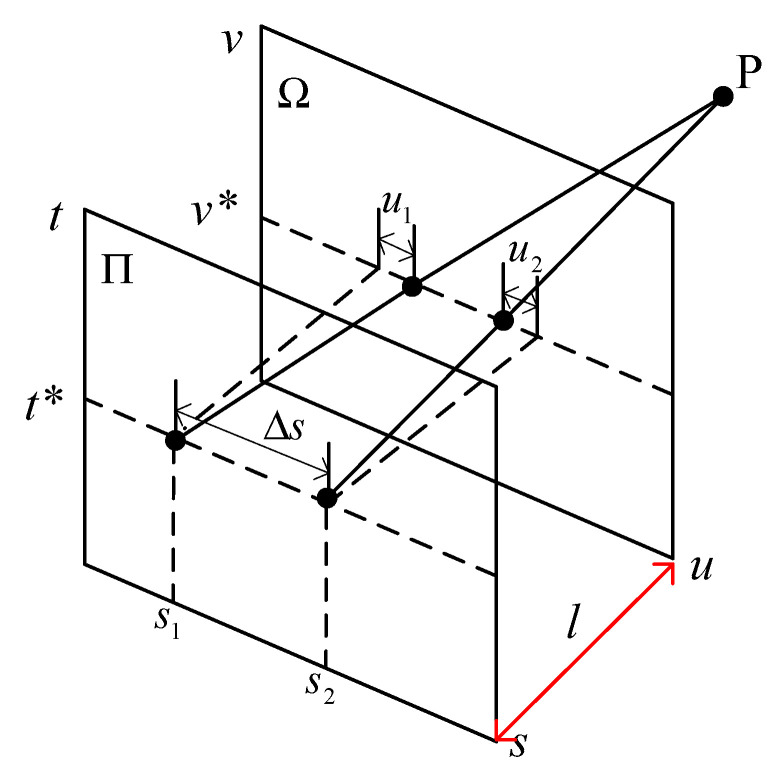
Light-field presentation using 2PP.

There are many ways to represent the light field, among which the two-plane parametrization (2PP) is very intuitive and commonly used. 2PP representation considers the light field as a collection of pinhole views from several viewpoints parallel to a common image plane. In this way, a 4D light field is defined as the set of rays on a ray space ℜ, passing through two planes Π and Ω in 3D space; as shown in Figure 1, the 2D plane Π contains the viewpoints given by (*s*,*t*), and Ω denotes the image plane parameterized by the coordinates (*u*,*v*). Therefore, each ray can be uniquely identified by intersections (*u*,*v*) and (*s*,*t*) with two planes. A 4D light field can be formulated as a map:

(1) L:Ω×Π→ℜ,(u,v,s,t)↦L(u,v,s,t)

An EPI can be regarded as a 2D slice of a 4D light field. If the coordinate *v* in the image plane Ω is a constant v*, and the coordinate *t* in the image plane Π keeps the value of t*, we will get a horizontal slice Sv*,t* of 4D light field, parameterized by coordinates *u* and *s*, that is

(2) $$S_{v*,t*}:(u,s)\to L(u,v*,s,t*)$$

In a 4D light field, an image under fixed viewpoint coordinates (s*,t*) is called a sub-aperture image $I_{s*,t*}$, as shown in Formula (3). If (s*,t*) is the center of all viewpoints, the image is also called the central view image. The sub-aperture image from the light field is similar to the scene image captured by a monocular camera.

(3) $$I_{s*,t*}:(u,v)\to L(u,v,s*,t*)$$

**Figure 2:**
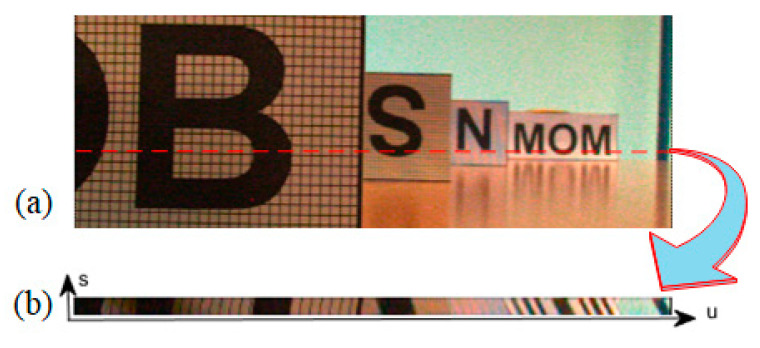
An example of an EPI. (**a**) is a central view image, (**b**) is an EPI corresponds to the row of the red dotted line in (**a**).

Figure 2 shows an example of a central view image and an EPI, where (a) is the central view image of the scene and (b) is the EPI in the horizontal direction. When we generate the EPI of Figure 2b from the light field data, the coordinate *v* is fixed at the position of the red dotted line in Figure 2a. In other words, the EPI of Figure 2b corresponds to the row of the red dotted line in Figure 2a. In Figure 2, the width of the EPI is the same as that of the central view image, and its height depends on the angular resolution of the light field (i.e., the range of the coordinate s).

The EPI shown in Figure 2 presents a distinct linear texture. It has been proved that the slopes of straight lines in an EPI contain depth information [10]. Let us consider the geometry of the map expression (2). In the set of rays emitted from a point *P*(*X*, *Y*, *Z*), the rays whose coordinates are (*u*,v*,*s*,t*) satisfy the geometric relationship shown in Figure 1, where v*, t* are constants and *u*, *s* are variables. According to the triangle similarity principle, the relationship between the image-point coordinates and the viewpoint coordinates conforms to Equation (4).

(4) $$\frac{\Delta s}{\Delta u}=-\frac{Z}{l}$$

In Equation (4), Δs and Δu signify the coordinate changes of viewpoint and image point respectively, where $\Delta s=s_{2}-s_{1}$, $\Delta u=u_{2}-u_{1}$; *Z* represents the depth of the point *P*, and *l* denotes the distance between two planes Π and Ω. 

Under the assumption of Lambert’s surface, the pixels corresponding to the same object point have the same gray level. These pixels with approximate gray values are arranged in a straight line when the 4D light field is transformed into 2D EPI. Equation (4) shows that the slope of the straight line is proportional to the depth of its corresponding object point. Therefore, the linear texture can be used as the geometric basis for depth estimation.

**Figure 3:**
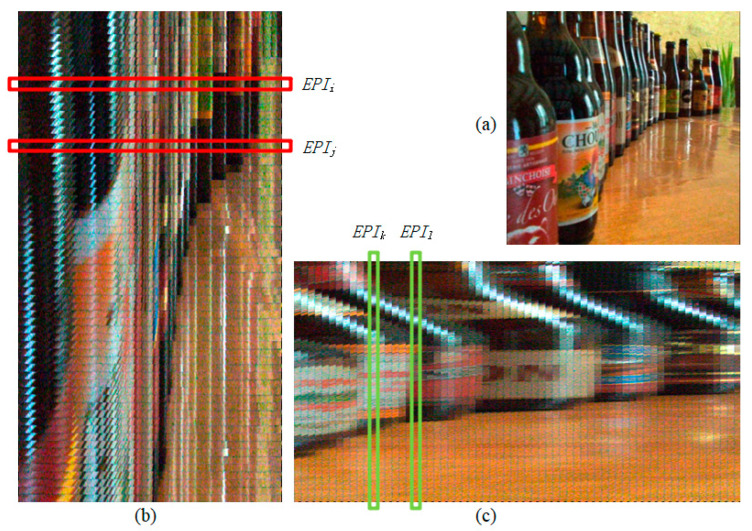
Examples of input images. (**a**) is a central view, (**b**) is a horizontal EPI synthetic image, (**c**) is a vertical EPI synthetic image.

Figure 2 only shows an EPI corresponding to one row in the central view image. In fact, each row position of the central view corresponds to its own EPI. Suppose we generate EPI for each row of the central view image, and stitch these EPIs one by one from top to bottom according to their corresponding row numbers in the central view image. In that case, we will get a horizontal EPI synthetic image abbreviated as EPIh for the whole scene. Figure 3 shows an example in which (a) is a central view image and (b) is part of a horizontal EPI synthetic image. *EPI_i_* and *EPI_j_* selected in Figure 3b represent EPI images corresponding to rows *i* and *j* in the central view image, respectively. These EPIs similar to *EPI_i_* are stitched from top to bottom to form a horizontal EPI synthetic image. It should be emphasized that Figure 3b is only a part of the vertical clipping of the whole EPIh so that the texture structure of the EPIh can be presented at a large display scale.

Similarly, in formula (2), if the coordinates *u* and *s* remain unchanged, but the coordinates *v* and *t* change, we will obtain an EPI corresponding to a column of pixels in the central view image. Then EPIs of each column in the central view image can be stitched into a vertical EPI synthetic image (EPIv). Figure 3c is part of a vertical EPI synthetic image, where the frames of *EPI_k_* and *EPI_l_* represent EPI images corresponding to columns *k* and *l* in the central view image, respectively.

It can be seen from the above synthesis process that the EPI synthetic images not only have local linear texture contained depth information but also integrate the spatial association information of the row or column in the central view image. Therefore, we use EPIh and EPIv as inputs of the deep neural network to improve feature extraction. Figure 3 illustrates some examples of these input images.

## 4. Network Architecture

Our deep neural network designed for depth estimation is described in this section. We first state the overall design ideas and outline the network architecture, followed by two structural details of our network, namely multi-stream inputs and skipconnections. Finally, we introduce the loss function used in training the network.

### 4.1. General Framework

We formulate depth estimation from the light field as a spatially dense prediction task, and design a deep convolution neural network (ESTNet) to predict the depth of each pixel in the central view image.

**Figure 4:**
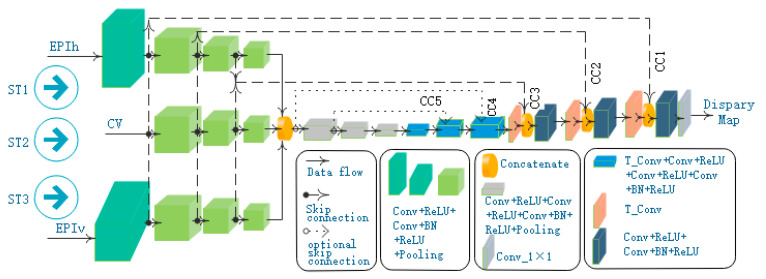
The architecture of ESTNet.

In general, the proposed model is a convolutional network with multi-stream inputs and some skip-connections shown in Figure 4. In essence, our network is a two-stage network. The first stage conducts a downsampling task, and the second stage performs an upsampling job. The downsampling part encodes input images in a lower dimensionality, while the upsampling part is designed to decode feature maps and produce dense predictions of each pixel.

In the downsampling stage, a multi-stream architecture is utilized to learn the geometry information of the light field. The main idea behind the multi-stream architecture is to receive different inputs from light-field data and extract and fuse their features. Three streams are designed with the respective input of the horizontal EPI synthetic image (EPIh), the central view image (CV), and the vertical EPI synthetic image (EPIv). Different from EPInet [5], EPIh and EPIv are fed with EPI synthetic images rather than the stack of the sub-aperture images in one direction. CNN is used to encode features in each stream. Then the outputs of these streams are concatenated for further encoding of features.

In the upsampling stage, the transposed convolution layer is used as the core of the decoding module to improve the resolution of the feature map. Besides, the connections between modules with the same resolution in the downsampling stage, and the upsampling stage are established to fuse the lower texture information and the upper semantic information. For the sake of the single-channel disparity map computation, a 1×1 convolution layer is added at the end of the network.

### 4.2. Multi-Stream Architecture

As shown in Figure 4, our model has three feature extraction streams: ST1, ST2, and ST3, which are fed with EPIh, CV, and EPIv, respectively.

The stream of ST1 is composed of four blocks with similar structures. Each block is a stack of convolutional layers: the first convolutional layer is followed by activation of ReLU, a batch normalization (BN) operation is executed after the second convolutional layer and provides input for a ReLU activation, and the end of each block is a max-pooling layer. The block structures in ST2 and ST3 are the same as those in ST1. In addition, ST3 contains the same number of blocks as ST1, and ST2 has only three blocks.

Now we discuss the size of input images for the three streams. Suppose the dimensions of the light field are $\left(N_{ar},N_{ac},N_{sr},N_{sc},N_{ch}\right)$, where $N_{ar},N_{ac}$ are angular resolution in a row and a column direction, respectively; $N_{sr},N_{sc}$ indicate space resolution in a row and a column direction, respectively; $N_{ch}$ represents the number of channels in a light-field image. EPIh generated from light-field data has the dimension of $\left(N_{ac}\times N_{sr},N_{sc},N_{ch}\right)$, the dimension of EPIv is $\left(N_{sr},N_{ar}\times N_{sc},N_{ch}\right)$, and the size of CV is $\left(N_{sr},N_{sc},N_{ch}\right)$. For example, the images in Figure 3 were generated from the (9,9,381,381,3) dimensional light-field data collected by the Lytro camera. The resolution of the central view image (Figure 3a) is 381 × 381, and the resolution of EPIh should be 3429 × 381. However, Figure 3b is only a part of EPIh, and its resolution is 1450 × 381.

As mentioned above, the input images in the three streams have different dimensions. However, the output of each stream should reach the same resolution for concatenation processing. Therefore, the parameters, such as the size and the stride for convolutional kernel or max pooling, should be set reasonably.

In the first block of the ST1 stream, as shown in Table 1, the first convolutional layer filters the $\left(N_{ac}\times N_{sr},N_{sc},N_{ch}\right)$-dimensional EPIh with 10 kernels of size (3,3) and a stride of 1 pixel. The second convolutional layer also has 10 kernels of size (3,3) and 1-pixel stride, followed by batch normalization (BN) layer and ReLU activation. The end of the first block is spatial pooling carried out by the max-pooling layer. Max-pooling is performed over a (9,1) pixel window, with stride (9,1). The first block of the ST3 stream is of similar structure as ST1′s first block, but ST3′s max-pooling stride is (1,9).

After the first block processing of the ST1 stream, its output resolution is consistent with that of the CV image. The same is true for the first block of the ST3 stream. Therefore, the identical layer structure is designed for the remaining three blocks in ST1 and ST3 streams and the blocks in ST2 stream. In these blocks, all the convolutional layers have kernel size of (3,3) and stride of 1 pixel, and a Max pooling layer use (2,2) window to slide with stride 2. The convolutional layers in one block have the same number of filters and the exact size of feature maps. However, from one block to the next, the feature map size is halved; the number of filters is doubled to preserve the time complexity per layer.

After the feature extraction of the three streams, we cascade their output results and then employ three blocks to extract features further. These blocks are shown in the gray box in Figure 4, where each block is composed of two Conv + ReLU layers, one Conv + BN + ReLU layer, and one max pooling layer.

After the above encoding stage of feature extraction, the network enters an expansive path that decodes the feature maps. This stage of the network consists of six blocks which are divided into two types. The first type block includes one transposed convolution layer, two Conv + ReLU layers, and one Conv + BN + ReLU layer. Table 2 lists the parameters of the first type block. Compared with the first type block, the second type block adds a cascade layer to realize skip-connections and reduce a Conv + ReLU layer. Finally, we use a 1 × 1 convolutional layer to get the disparity map.

### 4.3. Skip Connections

Compared with high-level feature maps, shallow features have smaller receptive fields and therefore contain less semantic information, but the image details are preserved better [26]. Since depth estimation requires both accurate location information and precise category prediction, fusing shallow and high-level feature maps is a good way to improve depth estimation accuracy. Therefore, the proposed model utilizes skip-connections to retain shallow detailed information from the encoder directly.

Skip-connections connect neurons in non-adjacent layers in a neural network. As shown in Figure 4, dotted lines indicate skip-connections. With those skip-connections in a concatenation fashion, local features can be transferred directly from a block of the encoder to the corresponding block of the decoder.

In Figure 4, CC1, CC2, and CC3 are the three skip-connections proposed in this paper. In order to analyze the impact of the number of skip-connections on our network performance, we compared the experimental results when adding CC4 and CC5 in the experimental section. The shallow feature map is directly connected to the deep feature map, which is essentially a cascade operation, so it is necessary to ensure that the resolution of the two connected feature maps is equal. In theory, skip-connections can be established for blocks with the same resolution in the two stages, but the experiment shows that three skip-connections can achieve better results.

### 4.4. Loss Function

The intuitive meaning of the loss function is obvious: the worse the performance of the model, the greater the loss, so the value of the corresponding loss function should be larger. When training a network model, the gradient of the loss function is the basis of updating network parameters. Ideally, the large value of the loss function indicates that the model does not perform well, and the gradient of the loss function should be large to update the model parameters quickly. Therefore, the selection of loss function affects the training and performance of the model.

We try to train the proposed network (ESTNet) with the loss function of log-cosh. Log-cosh is calculated by the logarithm of hyperbolic cosine of prediction error, as shown in Formula (5), where $y_{i}$ and $y_{i}^{p}$ refer to the ground-truth value and the prediction value respectively, and the subscript i represents the pixel index.

(5) $$L(y,y^{p})=\sum_{i=1}^{n}\log(\cosh(y_{i}^{p}-y_{i}))$$

The loss function of log-cosh is usually applied to regression problems, and its central part log(cosh(x)) has the following characteristics: if the value of x is small, it is approximately equal to $x^{2}/2$, and while x is large, it is close to (|x|−log(2)). This means that log-cosh works much like mean square error (MSE), but is not easily affected by outliers.

## 5. Experimental Results

### 5.1. Experimental Setup

#### 5.1.1. Dataset

Our model training is carried out on HCI Benchmark, and the evaluation is respectively conducted on HCI Benchmark [27] and the real light field dataset [28].

HCI benchmark has 28 scenes, each with 9 × 9 angular and 512 × 512 spatial resolutions. This benchmark is designed to include issues that are particularly challenging for the depth estimation procedure: occlusion of the boundaries, presence of structures, low textures, smooth surfaces, and camera noise. The scenes were created with Blender using the internal renderer for the stratified scenes and the Cycles renderer for the photorealistic scenes. The light field images are set up in a way such that all cameras are shifted towards a common focal plane while keeping the optical axes parallel. Most scene content lies within a range of −1.5 px and 1.5 px, though disparities on some scenes are up to 3 px. For each scene, HCI provides 8-bit light fields (9 × 9 × 512 × 512 × 3), camera parameters, and disparity ranges. For the stratified and training scenes, the benchmark further includes evaluation masks and 16bit ground truth disparity maps in two resolutions (512 × 512 px and 5120 × 5120 px).

The real light field dataset provided by Mousnier et al. [28] is used for testing. The dataset contains 30 groups of Lytro camera data, including 25 groups of indoor and outdoor scenes, three groups of motion blur, one group of long-time exposure, and one group of plane photography. The last three kinds of light-field images are not in the evaluation scope of the proposed method. This experiment mainly tests 25 light-field images of indoor and outdoor scenes.

#### 5.1.2. Experimental Scheme

The proposed network model is implemented using Keras with Tensorflow as the backend. Our experiment is conducted on hardware configured with Intel Xeon E5-2650 CPU, 64GB memory, and an NVIDIA Quadro K5200 GPU graphics card.

We use 16 scenes in the additional module of the HCI dataset for network training and 12 scenes in the structured, test, and training modules for network evaluation. In order to ensure that there are enough training images, we augment data by rotating light-field images to 90°, 180°, and 270° and flipping them. For the sake of reducing the memory consumption during training the network, the input of the network is a sub-image with only 64 × 64 resolution randomly selected from the 512 × 512 resolution image. Moreover, when preparing the network inputs, we do not rotate the entire 4D light field to generate a batch of enhanced sample data by slicing the new light field. Instead, we calculate a batch of enhanced samples through the designed algorithm, keeping the entire light-field data unchanged. Through the above measures, the number of training images is up to one million times the number of scenes, ensuring the sample diversity for network input.

We conduct a series of comparative experiments on HCI and real light-field datasets to evaluate our algorithm’s performance and verify the validity of the multi-stream and skip-connection network structure.

### 5.2. Evaluation Metrics

The hierarchical sense of the depth map, especially the evaluation of the object boundary, is of more concern. In our experiment, MSE and BadPix are selected as the evaluation metrics of algorithm performance analysis. These two metrics are described in reference [27] and are used by many current relevant methods. For the sake of clarity, we report this as follows.

Given an estimated disparity map d, the ground truth disparity map gt and an evaluation mask M, MSE is quantified as

(6) $$MSE_{M}=\frac{\sum_{x\in M}{\left(d\left(x\right)-gt\left(x\right)\right)}^{2}}{\left|M\right|}\times 100$$

And BadPix is formulated as

(7) $$BadPix_{M}\left(t\right)=\frac{\left|\left\{x\in M:\left|d\left(x\right)-gt\left(x\right)\right|>t\right\}\right|}{\left|M\right|}$$

In (7), t is a disparity error threshold, usually set to one value of 0.01, 0.03, and 0.07.

### 5.3. Ablation Studies

#### 5.3.1. Multi-Stream Architecture

To verify the effectiveness of the proposed multi-stream architecture, we cut down the network input streams and conduct experiments to evaluate the performance of single-stream, double-stream, and three-stream architecture, respectively. Figure 4 shows a three-stream network. In this network, deleting ST1 or ST3 leads to a double-stream network; a single-stream network is obtained if both ST1 and ST3 are deleted.

For the above networks with different structures, we train each network on the same dataset and keep the hyperparameters such as batch and epoch consistent. Then, each network is tested on the stratified and training groups in the HCI benchmark. According to the test results, Table 3 lists the average values of computational time, RMS, and BadPix in eight scenes of the HCI benchmark.

It can be seen from Table 3 that the network with three-stream architecture achieves the best effect, while the single-stream network is the worst. In the training of the single-stream network, the loss function reaches the expected threshold value quickly. However, when the network is applied to the test scene, the estimating depth at the object boundary is not ideal. This phenomenon may be because the network parameters are relatively small, and the EPI texture information is not fully utilized.

#### 5.3.2. Skip-Connection Architecture

In order to verify the structure of skip-connections, we designed four groups of experiments on four kinds of network, including no skip-connection (N-S0), one skip connection (N-S1), three skip-connections (N-S3), and five skip-connections (N-S5). Referring to Figure 4, we describe the structure of the four networks as follows. N-S0 network means that all skip-connections of CC1, CC2, CC3, CC4 and CC5 are deleted; if only CC1 is retained and the other skip-connections are deleted, N-S1 network is obtained; N-S3 network includes CC1, CC2, CC3, but not CC4 and CC5; N-S5 network includes all skip-connections in the figure.

Similar to the experimental analysis of multi-stream design, the above four networks (N-S0, N-S1, N-S2, N-S3, N-S4) are trained and tested on the HCI benchmark, respectively. Table 4 shows the evaluation results of each network regarding MSE and BadPix. As those data in Table 4 revealed, with the increase of skip-connections, the performance gradually improves. However, compared with the N-S3 network, the MSE of the N-S4 network is only slightly improved, but the BadPix is unchanged.

After the ablation study, we determine our network, including three input streams of ST1, ST2, ST3, and three skip-connections of CC1, CC2, and CC3 (see Figure 4).

### 5.4. Comparison to State-of-the-Art

#### 5.4.1. Quantitative Comparison

In this subsection, the performance of the proposed model is evaluated using a synthetic light-field dataset, namely the HCI dataset mentioned above. We compared our ESTNet with the following state-of-the-art models: EPInet [5], EPI-refocus-net [23], FunsionNet [24], and VommaNet [25]. These networks are trained and evaluated quantitatively based on the scenes and the ground truth values of the HCI dataset.

**Figure 5:**
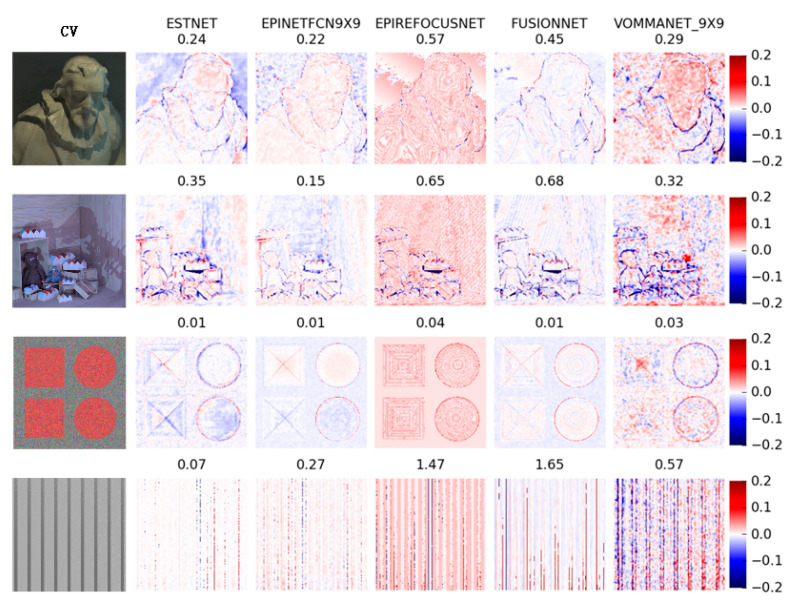
Examples of MSE results for each network.

Figure 5 shows the MSE results of each network in some scenes of HCI. Each row in the figure corresponds to a scene in the HCI dataset. The central view images of these scenes are arranged in the first column. The scene names are cotton, dino, pyramids, and stripes, from top to bottom. The other columns are the results of each network. The number above each image is the MSE metric value of the corresponding network in the scene. At the end of each line is the color ruler of the image. The smaller the deviation between the estimated depth and the true value of each pixel position in the image, the lighter the pixel’s color. Furthermore, the greater the positive deviation, the darker the red, the greater the negative deviation, and the darker the blue.

**Figure 6:**
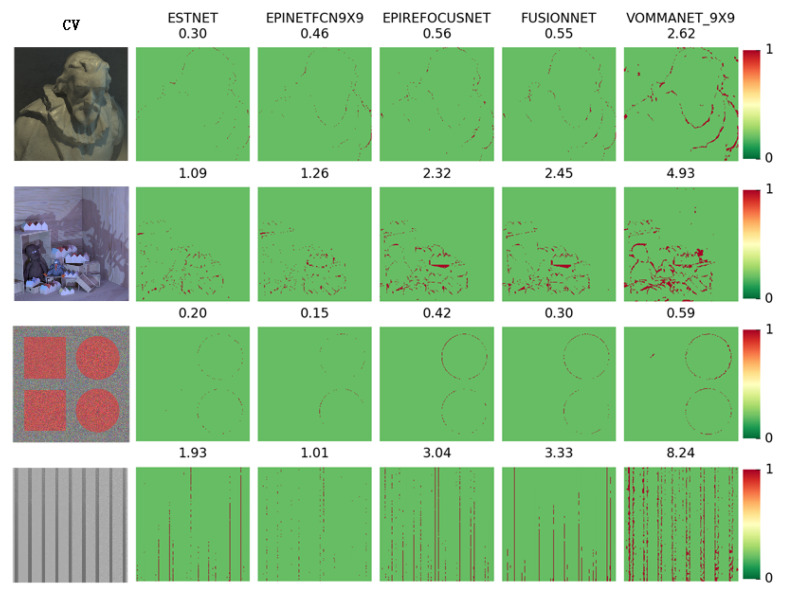
Examples of BadPix results for each network.

Figure 6 shows the Badpix index test results of each network. The layout of the figure is similar to that of Figure 5, but the color distribution of pixels is slightly different. When the Badpix error is small, the pixel is green, but when the Badpix error increases, the pixel changes from green to red.

It can be seen from Figure 5; Figure 6 that ESTNet proposed by this paper has achieved reasonable performance. Although our network is not the best in every scene, it has reached a better average level (see Table 5). As shown in Figure 5, the results of ESTNet show that the color in the beard of the cotton scene, the wall of the scene, the ball of the pyramids scene, and the stripe of the stripes scene tends to be white, indicating that the MSE index of these regions is small. Therefore, our network has a relatively excellent MSE index in the homogeneous region of the scene, which proves that our network can improve the accuracy of depth estimation by constructing EPI synthetic texture images.

As shown in Figure 5, the results of the current light field depth estimation methods based on neural networks generally exist in the case of relatively large MSE values at the object boundary. However, as seen in the Badpix index of Figure 6, the performance of our method is fairly good at the occlusion boundary. It shows that the depth estimation error of object boundary is larger than that of the internal area, but is still in the acceptable range (less than 0.07). Therefore, our network has some advantages in occlusion processing.

Table 5 lists the average MSE, average Badpix, and average computational time of each network when tested on the HCI dataset. These results come from the reports of each network author. Although in the examples of Figure 5; Figure 6, our ESTNet does not have the best metrics in every scene, it achieves the best average MSE and average BadPix metrics, as shown in Table 5.

The hardware platforms of each network are different (see Table 6), so the computational time cannot be strictly compared, but the difference in computational time of each network can still be analyzed according to the calculation power of the platform. Among K5200, TITAN X and 1080Ti, 1080Ti has the strongest computing power, but a single 1080Ti is less than two TITAN X and K5200 has the weakest computing power. Under the weakest GPU computing power, our ESTNet achieves the shortest computing time, even several orders of magnitude lower than EPI-refocus-net and FusionNet. Although the measurement of MSE and BadPix of our ESTNet is inferior to that of EPINet in some scenes, it has apparent advantages in computational time. To sum up, ESTNet can balance the accuracy of depth estimation and computational time.

#### 5.4.2. Qualitative Comparison

In this section, the experiments are carried out on the real light-field dataset to verify our ESTNet performance in the scenes with noise. As mentioned earlier, the Lytro camera captured this light field dataset [27], mixed with various imaging noises, but without depth ground-truth value. Therefore we make qualitative comparisons through visual observation.

**Figure 7:**
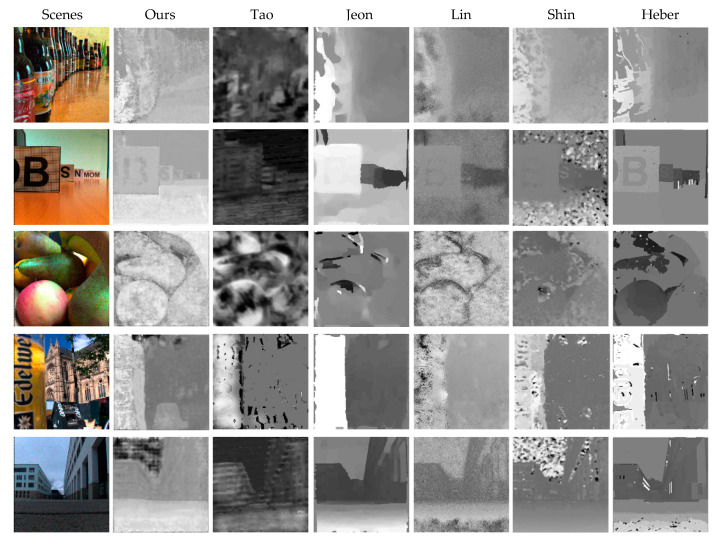
Examples of depth estimation results for each method in the real light field.

Considering the availability of method code, we choose Tao [7], Jeon [16], Lin [17], Shin [5], and Heber [22] methods to compare with our ESTNet. As shown in Figure 7, taking five scenes in the real light-field dataset as examples, the depth estimation results of each method are given in the columns.

Among methods of qualitative comparison experiment, Tao [7], Jeon [16], and Lin [17] are traditional methods based on energy optimization, while Shin [5], Heber [22], and ours are deep neural network methods. From the perspective of depth level, it can be seen from Figure 7 that the results of the methods based on deep neural networks are generally better than those of traditional energy optimization methods. The disparity maps of the traditional methods can only clearly show fewer depth levels. In particular, the results of the Tao method are fuzzy, and the contour of the object is invisible. On the contrary, the results of Shin, Heber, and our network can show more depth levels.

The method based on depth neural network generally has insufficient smoothness; especially, the EPINet results show more noise patches in the flat area. However, the results of traditional methods are smoother in homogeneous regions because the energy function used by the traditional method contains the smoothing term.

It is worth mentioning that our network has achieved relatively good depth estimation results. For instance, in the scene shown in the last row of Figure 7, the sky is inverted at a little distance by all other methods. But our approach successfully deals with the depth level of the sky.

These methods use different programming languages and different platforms. Some methods call on GPU resources, while others only use CPU. Therefore, the computational time is not comparable and is not listed.

## 6. Conclusions

In this paper, ESTNet is designed for light-field depth estimation. The idea behind our design is the principle of epipolar geometry and the texture extraction ability of a convolutional neural network (CNN). We first analyze the proportional relationship between the depth information and the slope of the straight line in an EPI, and then combine EPIs by row or column to generate EPI synthetic images with more linear texture. The proposed ESTNet network uses multi-stream inputs to receive three kinds of image with different texture characteristics: horizontal EPI synthetic image (EPIh), central view image (CV), and vertical EPI synthetic image (EPIv). EPIh and EPIv have more abundant textures suitable for feature extraction by CNN. ESTNet is an encoding-decoding network. Convolution and pooling blocks encode features, and then the transposed convolution blocks decode features to recover depth information. Skip-connections are added between encoding blocks and decoding blocks to fuse the shallow location information and deep semantic information. The experimental results show that EPI synthetic images as the input of CNN are conducive to improving depth estimation performance, and our ESTNet can better balance the accuracy of depth estimation and computational time.

Since the strides of the first max-pooling layers of ST1 and ST3 in ESTNet are (9,1) and (1,9), respectively, the limitation of our method is that the number of views used in EPI synthetic images is fixed to nine. At present, the number of horizontal and vertical views of most light field cameras is nine or more. If there are more than nine views in the horizontal or vertical direction, we can select only nine. Therefore, although this method cannot be adaptive to the number of views, it can estimate depth from light-field data captured by most plenoptic cameras available.

## Figures and Tables

**Table 1:** The first block structure in ST1 stream.

Layer	Type	Output Shape	Parameters
ST1_g1c1	Conv2D	(None,4608,512,10)	Kernel_size = (3,3), stride = 1, filter_num = 10
ST1_g1r1	Activation	(None,4608,512,10)	
ST1_g1c2	Conv2D	(None,4608,512,10)	Kernel_size = (3,3), stride = 1, filter_num = 10
ST1_g1BN	Batch Normalization	(None,4608,512,10)	
ST1_g1r2	Activation	(None,4608,512,10)	
ST1_g1p	Max Pooling	(None,512,512,10)	Pool_size = (1,9)

**Table 2:** The first block structure in the decoding stage.

Layer	Type	Output Shape	Parameters
D_dec_1	Conv2DTranspose	(None,64,64,80)	Kernel_size = (2,2), stride = (2,2), filter_num = 80
D_c1_1	Conv2D	(None,64,64,80)	Kernel_size = (2,2), stride = (2,2)
D_relu1_1	Activation	(None,64,64,80)	
D_c2_1	Conv2D	(None,64,64,80)	Kernel_size = (2,2), stride = (2,2)
D_relu2_1	Activation	(None,64,64,80)	
D_c3_1	Conv2D	(None,64,64,80)	Kernel_size = (2,2), stride = (2,2)
D_BN1_1	Batch Normalization	(None,64,64,80)	
D_relu3_1	Activation	(None,64,64,80)	

**Table 3:** Performance comparison of networks with the different number of input streams.

Type	Computational Time (Avg. Units: s)	MSE (Avg.)	BadPix(0.07) (Avg.)
ST1	0.1914	6.72	12.85
ST2	0.1905	10.04	18.93
ST3	0.1914	6.75	12.87
ST1 + ST2	0.2235	4.48	9.32
ST2 + ST3	0.2235	4.51	9.41
ST1 + ST2 + ST3	0.2314	1.65	3.86

**Table 4:** Performance comparison of networks with the different number of skip-connections.

Type	MSE (Avg.)	BadPix(0.07) (Avg.)
N-S0	8.59	10.25
N-S1	3.64	6.85
N-S2	2.85	5.02
N-S3	1.65	3.86
N-S4	1.60	3.81
N-S5	1.60	3.80

**Table 5:** Performance comparison of current popular neural networks.

Methods	Computational Time (Avg. Units: s)	MSE (Avg.)	BadPix(0.07) (Avg.)
ESTNet	0.2314	1.652	3.857
EPINet_9 × 9 [5]	2.041	2.521	5.406
EPI-refocus-net [23]	303.757	3.454	5.029
FusionNet [24]	303.507	3.465	4.674
Vommanet_9 × 9 [25]	1.882	2.556	10.848

**Table 6:** Runtime environment of current popular neural networks.

Methods	Runtime Environment
ESTNet	Window 10 64bit, Intel Xeon E5-2650 @2.3GHz, 64GB RAM, NVIDIA Quadro K5200 GPU
EPINet_9 × 9 [5]	Window10 64bit, i7-7700 @3.6GHz, 32GB RAM, 1080Ti
EPI-refocus-net [23]	ntel Core i7-4720HQ 2.60GHz + Two TITAN X GPUs
FusionNet [24]	Intel Core i7-4720HQ 2.60GHz + Two TITAN X GPUs
Vommanet_9 × 9 [25]	Ubuntu 16.04 64bit, E5-2603 v4 @1.7GHz 64GB RAM, GTX 1080Ti

## Data Availability

HCI benchmark [27]: https://lightfield-analysis.uni-konstanz.de (accessed on 30 August 2021); Real light-field dataset [28]: Available online: http://www.irisa.fr/temics/demos/lightField/index.html (accessed on 30 August 2021).
